# Analytical Model of Hole Diameter and Self-Guiding Machining Mechanism of BTA Deep Hole Drilling

**DOI:** 10.3390/ma15155329

**Published:** 2022-08-02

**Authors:** Xubo Li, Jianming Zheng, Biao Yu, Yongqiang Du, Yanan Zhou

**Affiliations:** 1 College of Mechanical Engineering, Baoji University of Arts and Sciences, Baoji 721016, China; biaoyu0213@163.com (B.Y.); dyq2022617@163.com (Y.D.); zyn1234560627@163.com (Y.Z.); 2 Shaanxi Key Laboratory of Advanced Manufacturing and Evaluation of Robot Key Components, Baoji 721016, China; 3 School of Mechanical and Precision Instrument Engineering, Xi’an University of Technology, Xi’an 710048, China; zjm@xaut.edu.cn

**Keywords:** deep hole drilling, BTA drill, extrusion contact, FEM simulation, analytical model

## Abstract

The goal of this study was to explore the self-guided machining mechanism of boring and trepanning association (BTA) deep hole drilling and realize precise control of the machining quality. The motion analysis method was used to analyze the center motion trajectory of the drill during the entrance, and the self-guiding mechanism and hole-forming mechanism of BTA deep hole drilling were revealed. Considering the bending deformation of the drilling tube and the tool structure parameters, according to the elastic-plastic deformation theory and Hertzian contact theory, a novel analytical model of the extrusion contact between the guide pads and the hole wall of the BTA deep hole drilling was established for the theoretical prediction of the extrusion deformation and the machining hole diameter. Combined with the finite element method (FEM) simulation model, the variation law of the contact inclination angle, contact stress, and extrusion deformation of the guide pads and the hole wall with the drilling conditions were studied. The total extrusion deformation between the guide pad and the hole wall was between 10 and 50 μm. The maximum error between the FEM simulation results and the test results was 18.1%, and the maximum error between the analytical model results and the test results was 23.6%. The simulation and experimental results showed that the established extrusion contact model could accurately predict the extrusion deformation of the hole wall and the machining hole diameter.

## 1. Introduction

With the rapid and vigorous development of the global manufacturing industry, the demand for precision deep hole machining technology in the fields of automobiles, energy, weapons, aerospace, and the chemical industry is increasing [1,2,3]. Boring and trepanning association (BTA) deep hole drilling is a high-efficiency internal chip removal self-guided machining method for drilling deep holes with a large length–diameter ratio. Compared with gundrilling, the tube of BTA deep hole drilling has higher strength, and a larger drilling feed can be selected. At the same time, the internal chip removal method is adopted to avoid the scratches between the chip and the machined hole wall, and the machined hole wall has a better surface finish [4,5].

However, the structure of the BTA drill is complex, and the rigidity of the deep hole machining tool system is poor. BTA deep hole machining is a composite-forming method of tooth cutting and guide pad extrusion. The guide pads balance the tooth cutting force, and secondary ironing on the forming hole walls of the tooth cutting is performed to realize the self-guiding of deep hole machining, thereby ensuring machining hole accuracy [6,7,8]. The mechanism of self-guiding deep hole drilling is extremely complex, which makes it difficult to accurately control the quality of BTA deep hole drilling [9,10,11,12].

In order to improve the quality of BTA deep hole machining, some scholars have studied the influence of the tool cutting edge angle. Tnay et al. [13] studied the effect of the approach angle of the deep hole drilling tool cutting edge on chip removal efficiency and tool wear. Zhang et al. [14] reported the influence of the vertex offset of the inner and outer gundrilling tool cutting edges on the deep hole straightness. The quality of deep hole machining is affected not only by the tooth cutting, but also by the extrusion of the guide pads and the hole wall. In BTA deep hole machining, the extrusion deformation of the guide pads and the hole wall affects the surface morphology and dimensional accuracy of the machined hole. A suitable extrusion contact state can not only reduce the roughness of the hole wall, but also decrease the contact stress between the guide pads and the hole wall, as well as slow down tool wear [15,16]. Woon et al. [17] found that the deflection of the machined hole is affected by the contact state between the guide pad and the hole wall. The extrusion of the guide pad to the hole wall causes a large deformation on the thin-walled side, and the axis of the deep hole becomes inclined toward the thin-walled side of the workpiece. Zhang et al. [18] showed that a larger extrusion deformation of the guide pads and the hole wall results in a larger hole diameter of deep hole drilling. Next, some scholars explored the structural parameters and position angles of the guide pads of the deep hole drilling tool. Felinks et al. [19] indicated the effect of the extruding chamfer of the guide pad on the vibration of the deep hole machining tool system. Neo et al. [20] illustrated the variation law of the hole wall roundness of deep hole gundrilling with guide pad wear and drilling depth. Yang et al. [21] studied the influence of the guide pad shape of the BTA drill on the vibration of the tool system and the temperature of the cutting zone. Astakhov [22,23] showed the influence of a fan-shaped guide pad and two strip-shaped guide pads on the drilling hole diameter. Matsuzaki et al. [24] explained the influence of the contact state between the guide pad and the hole wall on the defects of the rifling hole, and the surface quality of the machined hole could be improved by adding a third guide pad on the circumference of the body for a BTA drill. Sakuma et al. [25] analyzed the self-steering principle of deep hole drilling and the extrusion deformation of the guide pad to the hole wall, and they gave an approximate calculation method for the extrusion deformation, but did not consider the influence of the geometric parameters of the BTA drill and the deformation of the drilling tube. The extrusion state of the guide pad on the hole wall directly affects the machining accuracy of the deep hole and the surface quality of the hole wall. Only by exploring the self-guiding hole-forming mechanism of BTA deep hole drilling and establishing the extrusion contact model between the guide pads and the hole wall can the deep hole processing quality be accurately controlled [5,16].

Despite extensive work on the quality control of deep hole drilling, there are few reports on the self-guiding mechanism of BTA deep hole machining and the modeling of the extrusion contact between the guide pads and the hole wall. In this paper, on the basis of an analysis of the movement trajectory of the drill center during BTA deep hole drilling, the mathematical equations of the self-guiding hole-forming parameters are constructed. An analytical model of the extrusion contact between the guide pads and the hole wall of BTA deep hole drilling is established considering the structural parameters of the tool and the bending deformation of the drilling tube. With the help of finite element method (FEM) simulations and deep hole drilling experiments, the variation laws of contact inclination angle, contact stress, extrusion deformation, and machining hole diameter with drilling parameters in self-guiding hole forming are obtained, and the validity of the model is verified. This study can provide a theoretical basis for the optimized design of the BTA deep hole tool system and the quality control of precision deep hole machining.

## 2. Methodology

### 2.1. Hole-Forming Mechanism

**Figure 1:**
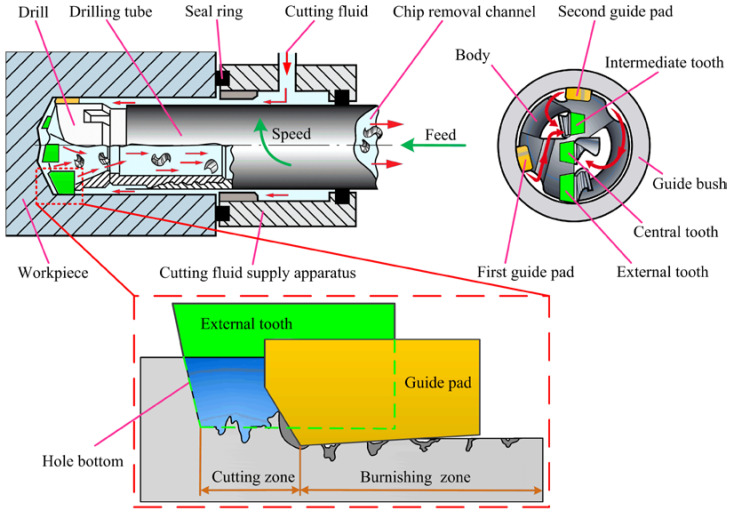
The principle of BTA self-guiding deep hole machining for internal chip removal.

The process of the BTA internal chip removal deep hole drilling system is shown in Figure 1. The BTA drill has three teeth in a dislocation distribution, and two guide pads are distributed on the outside of the drill body. The teeth are used to remove material and produce perfect chips, and high-pressure cutting fluid is used to remove chips from the drill cavity and hollow drilling tube. The length of the deep hole drilling tube is large, and the rigidity of the drilling tool system is weak. Guide pads are used to balance the cutting force of the teeth to realize the self-centering and guidance of the deep hole machining tool system. At the same time, the guide pads produce elastic–plastic extrusion to the hole wall, which improves the processing quality of deep hole drilling.

**Figure 2:**
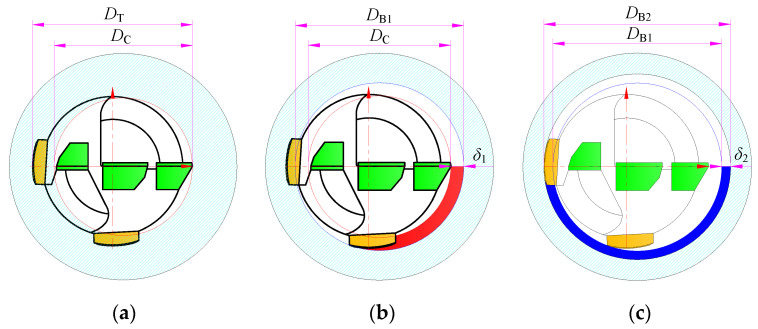
Self-guided hole forming process for BTA deep hole drilling: (**a**) hole formed by tooth cutting; (**b**) hole formed by extrusion of first guide pad; (**c**) hole formed by extrusion of second guide pad.

The BTA deep hole machining system can be divided into the tool rotary type, workpiece rotary type, and tool and workpiece double-rotary type. However, regardless of the machining method, the resultant force of the tooth cutting always presses the two guide pads against the hole wall. The self-guiding hole forming process of BTA deep hole drilling is regarded as having three stages: the tooth cutting hole-forming stage, the first guide pad extrusion hole-forming stage, and the second guide pad extrusion hole-forming stage, as shown in Figure 2.

In the hole-forming stage of tooth cutting, due to the offset of the drill rotation center relative to the machining hole center, the sizing edge of the external tooth is close to the hole rotation center, resulting in the hole diameter *D*_C_ formed by tooth cutting being smaller than the drill diameter *D*_T_.

In the first guide pad extrusion hole-forming stage, the hole wall formed by tooth cutting is extruded and deformed by the first guide pad. Assuming that the extrusion deformation of the first guide pad is *δ*_1_, the hole diameter *D*_B1_ formed by the extrusion of the first guide pad is enlarged by 2*δ*_1_ on the basis of the hole diameter *D*_C_ formed by tooth cutting.

In the second guide pad extrusion hole-forming stage, the hole wall formed by the first guide pad burnishing is extruded and deformed by the second guide pad. Assuming that the extrusion deformation of the second guide pad is *δ*_2_, the hole diameter *D*_B2_ formed by the extrusion of the second guide pad is enlarged by 2*δ*_2_ on the basis of the hole diameter *D*_C_ formed by the first guide pad burnishing.

However, elastic recovery occurs after the guide pad leaves the extrusion contact area. Assuming that the springback of the two guide pads and the hole wall after extrusion deformation are *δ*_e,1_ and *δ*_e,2_, respectively, the final diameter of the self-guided hole formed by the BTA deep hole drilling can be expressed as

(1) $$D_{H}=D_{C}+2\left(\delta_{1}+\delta_{2}\right)-2(\delta_{e,1}+\delta_{e,2})=2\left(R_{C}+\delta_{p,1}+\delta_{p,2}\right)$$

where *δ*_p,1_ and *δ*_p,2_ denote the plastic extrusion deformation of the first and second guide pads to the hole wall, respectively, and *R*_C_ is the cutting radius of tooth cutting.

**Figure 3:**
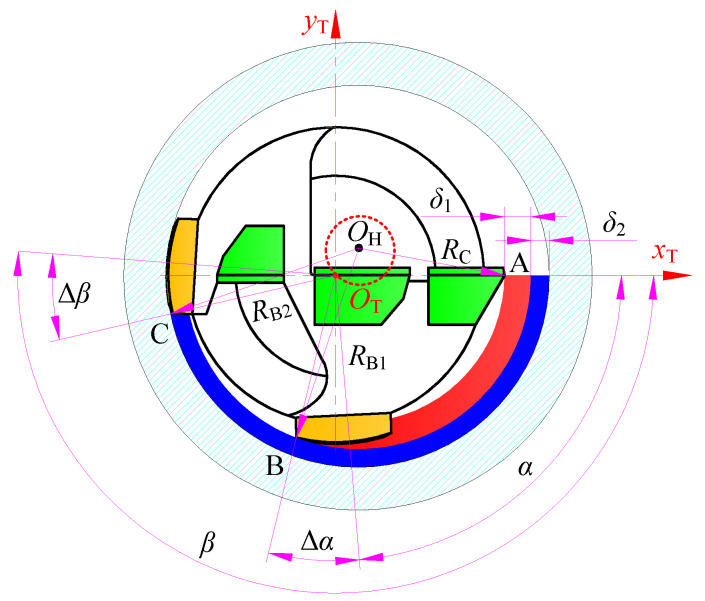
Geometric parameter relationship of staggered tooth BTA deep hole drilling.

After analyzing the motion trajectory of the BTA deep hole drilling tool and the self-guided hole-forming process, the geometric relationship between the cutting and extrusion deformation parameters during hole forming can be obtained, as shown in Figure 3. The interaction points of the drill and the hole wall are point A of the external tooth, point B of the first guide pad, and point C of the second guide pad. *R*_B1_ is the radius of the first guide pad extruded into the hole, whereas *R*_B2_ is the radius of the second guide pad extruded into the hole.

Assuming that the coordinate of the machining hole center *O*_H_ in the *x*_T_*O*_T_*y*_T_ coordinate system of the BTA drill is (*x*_O_, *y*_O_), and the radius of the drill is *R*_T_, then the coordinate of point A is (*R*_T_, 0), the coordinate of point B is (*R*_T_cos(*α* + Δ*α*), −*R*_T_sin(*α* + Δ*α*)), and the coordinate of point C is (*R*_T_cos(*β* − Δ*β*), −*R*_T_sin(*β* − Δ*β*)). Subsequently, the following relationships can be obtained:

(2) $$\{\begin{array}{l} R_{C}=\bar{AO_{H}}=\sqrt{{\left(x_{O}-R_{T}\right)}^{2}+y_{O}{}^{2}}\\ R_{C}+\delta_{1}=\bar{BO_{H}}=\sqrt{{\left(x_{O}-R_{T}\cos(\alpha +\Delta \alpha )\right)}^{2}+{\left(y_{O}+R_{T}\sin(\alpha +\Delta \alpha )\right)}^{2}}\\ R_{C}+\delta_{1}+\delta_{2}=\bar{CO_{H}}=\sqrt{{\left(x_{O}-R_{T}\cos(\beta -\Delta \beta )\right)}^{2}+{\left(y_{O}+R_{T}\sin(\beta -\Delta \beta )\right)}^{2}} \end{array}$$

where *α* and *β* are the position angles of the first and the second guide pads, respectively, and Δ*α* and Δ*β* are the included angles between the support points and the center lines of the two guide pads.

Combining Equations (1) and (2), the final hole diameter of BTA self-guided deep hole machining can be obtained as

(3) $$D_{H}=2\left(\sqrt{\begin{array}{l} {\left(\frac{\delta_{1}\left(\sin\left(\alpha +\Delta \alpha \right)-\sin\left(\beta -\Delta \beta \right)\right)+\delta_{2}\sin\left(\alpha +\Delta \alpha \right)}{\left(\sin\left(\alpha +\Delta \alpha \right)-\sin\left(\beta -\Delta \beta \right)\right)\left(\sin\left(\alpha +\Delta \alpha \right)+\sin\left(\beta -\Delta \beta \right)+1\right)}-R_{T}\right)}^{2}+\\ {\left(\frac{\delta_{1}\left(\cos\left(\beta -\Delta \beta \right)-\cos\left(\alpha +\Delta \alpha \right)\right)+\delta_{2}\left(1-\cos\left(\alpha +\Delta \alpha \right)\right)}{\left(\sin\left(\beta -\Delta \beta \right)-\sin\left(\alpha +\Delta \alpha \right)\right)\left(\sin\left(\alpha +\Delta \alpha \right)+\sin\left(\beta -\Delta \beta \right)+1\right)}\right)}^{2} \end{array}}+\delta_{p,1}+\delta_{p,2}\right)$$

### 2.2. Extrusion Deformation

**Figure 4:**
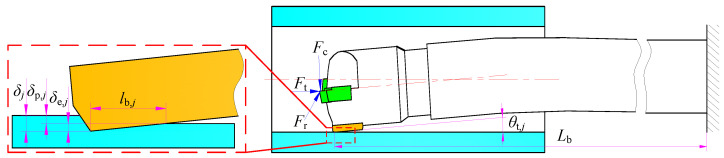
Extrusion deformation between the guide pads and the hole wall.

A schematic diagram of the extrusion contact deformation between the hole wall and the guide pads of BTA deep hole drilling is shown in Figure 4. Since there is a contact inclination angle between the guide pad and the hole wall, the guide pad will not be in contact with the hole wall as a whole along the axis direction. The extrusion deformation between the guide pad and the hole wall is the maximum deformation *δ_j_* before the guide pad leaves the hole wall and contacts the particle, which includes the plastic deformation *δ*_p,*j*_ and the elastic deformation amount *δ*_e,*j*_ of elastic recovery after the guide pad leaves, where *j* takes values of 1 or 2 to represent the number of the guide pad.

**Figure 5:**
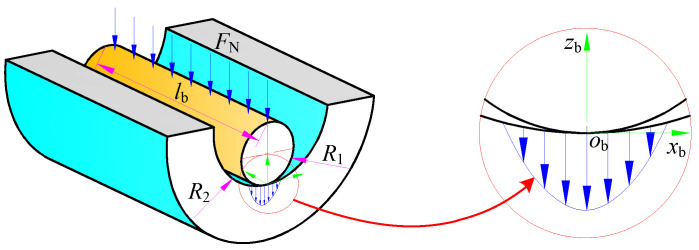
Hertz contact model of the guide pad and the hole wall.

Since the extrusion contact deformation between the guide pad and the hole wall is a small deformation, it conforms to the assumptions of the Hertzian contact theory. Hence, it can be considered as the extrusion contact problem of two cylinders inscribed along the axis direction [26]. The outer surface of the guide pad is regarded as the outer surface of the inner cylinder, and the hole wall formed by tooth cutting is regarded as the inner surface of the outer cylinder; their contact relationship is shown in Figure 5.

According to the Hertzian contact theory, the positive pressure *F*_N,*j*_ applied to the guide pad is proportional to the indentation depth *δ*_e,*j*_ of the hole wall.

(4) $$F_{N,j}=\frac{\pi }{4}E^{*}l_{b,}{}_{j}\delta_{e,j}$$

The maximum contact stress at the center *o*_b_ point of the contact area is

(5) $$\sigma_{0,j}={\left(\frac{F_{N,j}E^{*}}{l_{b,j}\pi R_{s}}\right)}^{\frac{1}{2}}$$

with

(6) $$E^{*}=\frac{1-\nu_{1}^{2}}{E_{1}}+\frac{1-\nu_{2}^{2}}{E_{2}}$$

(7) $$\frac{1}{R_{s}}=\frac{1}{R_{1}}-\frac{1}{R_{2}}$$

where *l*_b,*i*_ is the contact length between the guide pad and the hole wall along the axis direction, E* is the equivalent elastic modulus, *E*_1_ and *E*_2_ are the elastic moduli of the two contact bodies, *v*_1_ and *v*_2_ are Poisson’s ratios, 1*/R*_s_ is the relative curvature of the two contact bodies, *R*_1_ is the radius of the cylinder, and *R*_2_ is the inner surface radius of the elastic half-space.

Firstly, taking the first guide pad as the research object, it can be known from Equation (5) that the relationship between the positive pressure applied in the elastic range of the material and the maximum stress in the contact area is

(8) $$F_{N,1}=\frac{\sigma_{0,1}{}^{2}\pi R_{s}l_{b,1}}{E^{*}}$$

In the process of BTA deep hole drilling, the contact state between the guide pad and the hole wall is a complex compressive elastic–plastic contact deformation. Since the von Mises yield condition considers the influence of intermediate stress on the material yield, it is more in line with the actual working conditions [27]. Therefore, assuming that the plastic yield of the extrusion contact between the guide pad and the hole wall follows the von Mises yield condition, when the hole wall material reaches the elastic limit point, i.e., the plastic yield point, the positive pressure that needs to be exerted on the guide pad is

(9) $$F_{\mathrm{Ns},1}=\frac{3\tau_{s}{}^{2}\pi R_{s}l_{b,1}}{E^{*}}$$

By substituting Equation (9) into Equation (4), the extrusion deformation when the material of the hole wall begins to plastically yield can be obtained.

(10) $$\delta_{\mathrm{es},1}=\frac{12\tau_{s}{}^{2}R_{s}}{E^{*}{}^{2}}$$

As the radius of the contact surface between the guide pad and the hole wall is the same as the nominal radius of the drill, the actual contact width is narrow; thus, the guide pad with a width of *B*_p_ and an outer radius of *R*_T_ is equivalent to an elastic half-space body with a radius of *B*_p_/2 for elastic–plastic calculations. According to Equation (7), the equivalent radius of the cylinder for the elastic–plastic extrusion of the guide pad and the hole wall is

(11) $$R_{s}=\frac{R_{T}B_{p}}{2R_{T}-B_{p}}$$

In addition, the width of the front chamfer of the guide pad is small; hence, the influence of the chamfer on the contact length of the hole wall is ignored during modeling. The distance between the maximum point of extrusion deformation and the surface layer after extrusion is equal to the elastic recovery deformation *δ*_es,1_ reaching the plastic yield point of the material; then, the extrusion contact length between the guide pad and the hole wall along the axis direction is

(12) $$l_{b,1}=\frac{\delta_{\mathrm{es},1}}{\sin\theta_{t,1}}=\frac{12\tau_{s}{}^{2}R_{s}}{E^{*}{}^{2}\sin\theta_{t,1}}$$

where *θ*_t,1_ is the included angle between the first guide pad and the hole wall after contact deformation.

**Figure 6:**
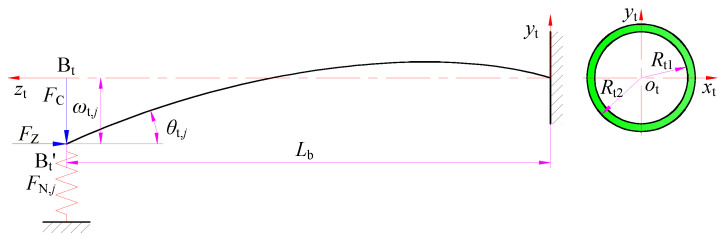
Bending deformation of the drilling tube in BTA deep hole drilling.

The rigidity of the BTA drill body is much greater than that of the drilling tube. Therefore, when studying the deformation of the BTA deep hole drilling tool system, the deformation of the body is ignored, and the body is regarded as a rigid body. The tool system can be thought of as a cantilever beam supported by springs. Considering that the drilling tube is always in a suspended state in the high-pressure cutting fluid during BTA deep hole drilling, the influence of the gravity of the drilling tube and the drill is ignored in the modeling. The cantilever beam is bent and deformed by the resultant cutting force *F*_C_ of the teeth, and it is also bent and deformed by the extrusion of the axial force *F*_Z_ in the opposite direction of the feed, as shown in Figure 6.

The deflection *ω*_t,*j*_ and the end-section rotation angle *θ*_t,*j*_ are produced by the bending moment at the end of the drilling tube. The differential equation of the deflection line of the drilling tube during the deep hole drilling is

(13) $$\frac{d^{2}\omega_{t,j}}{dz_{t}^{2}}=\frac{M_{t,j}}{E_{t}I_{t}}$$

with

(14) $$I_{t}=\frac{\pi \left(R_{t2}^{4}-R_{t1}^{4}\right)}{64}$$

where *M*_t,*j*_ is the bending moment of the drilling tube, *E*_t_ is the elastic modulus of the drilling tube, *I*_t_ is the inertia moment of the drilling tube, *L*_b_ is the suspended length of the drilling tube *R*_t1_ is the inner diameter of the drilling tube, and *R*_t2_ is the outer diameter of the drilling tube.

As a result of the bending deformation of the drilling tube in BTA deep hole drilling being small, for different types of loads, the deflection and end-section rotation angle can be calculated by superposition. Under the action of the cutting force *F*_C_, the positive pressures of the two guide pads are *F*_N,1_ and *F*_N,2_, respectively. The pressure of each guide pad along the bending and deformation directions of the drilling tube is equal to the reaction force of the positive pressure on the guide pads, while ignoring the influence of the bending deformation of the drilling tube on the direction of the drilling axial force. When the first guide pad is subjected to the positive pressure *F*_N,1_ and the drilling tube produces a bending moment *M*_tN,1_ = *F*_N,1_*L*_b_, the maximum deflection and end-section rotation angle of the drilling tube are as follows:

(15) $$\omega_{\mathrm{tN},1}=\frac{F_{N,1}L_{b}{}^{3}}{3E_{t}I_{t}}$$

(16) $$\theta_{\mathrm{tN},1}=\frac{F_{N,1}L_{b}{}^{2}}{2E_{t}I_{t}}$$

When the drilling tube generates torque *M*_tZ,1_ = *F*_Z_*ω*_tN,1_ under the action of the axial force *F*_Z_, the end-section rotation angle generated by the drilling tube is

(17) $$\theta_{\mathrm{tZ},1}=\frac{F_{Z}\omega_{\mathrm{tN},1}L_{b}}{E_{t}I_{t}}$$

Therefore, the contact inclination angle of the first guide pad can be obtained as

(18) $$\theta_{t,1}=\theta_{\mathrm{tN},1}+\theta_{\mathrm{tZ},1}+\alpha_{ct}=\frac{32F_{N,1}L_{b}{}^{2}\left(3\pi E_{t}\left(R_{t2}^{4}-R_{t1}^{4}\right)+128F_{Z}L_{b}{}^{2}\right)+3\pi^{2}E_{t}{}^{2}\left(R_{t2}^{4}-R_{t1}^{4}\right)\alpha_{ct}}{3\pi^{2}E_{t}{}^{2}\left(R_{t2}^{4}-R_{t1}^{4}\right)}$$

where *α_ct_* is the reverse taper angle of the guide pad along the axial direction.

In the same way, under the action of the pressure *F*_N,2_ and the axial force *F*_Z_, the contact inclination angle generated at the second guide pad is

(19) $$\theta_{t,2}=\frac{32F_{N,2}L_{b}{}^{2}\left(3\pi E_{t}\left(R_{t2}^{4}-R_{t1}^{4}\right)+128F_{Z}L_{b}{}^{2}\right)+3\pi^{2}E_{t}{}^{2}\left(R_{t2}^{4}-R_{t1}^{4}\right)\alpha_{ct}}{3\pi^{2}E_{t}{}^{2}\left(R_{t2}^{4}-R_{t1}^{4}\right)}$$

During the plastic extrusion deformation process between the guide pad and the hole wall, according to the assumption of the linearly strengthened elastic–plastic stress–strain model, combined with Equation (4), the relationship between the positive pressure exerted on the first guide pad and the extrusion deformation of the hole wall can be obtained as

(20) $$F_{N,1}-F_{\mathrm{Ns},1}=\frac{\pi }{2}E^{*}l_{b,1}\left(\delta_{1}-\delta_{\mathrm{es},1}\right).$$

According to Equation (20), the remaining plastic deformation after the elastic recovery of the extrusion deformation between the guide pad and the hole wall is

(21) $$\delta_{p,1}=\delta_{1}-\delta_{\mathrm{es},1}=\frac{2\left(F_{N,1}-F_{\mathrm{Ns},1}\right)}{\pi E^{*}l_{b,1}}.$$

Combining Equations (9)–(12) and Equation (21), the plastic deformation and total deformation of the extrusion contact between first guide pad and the hole wall can be obtained as

(22) $$\delta_{p,1}=\frac{F_{N,1}E^{*}{}^{3}{\left(2R_{T}-B_{p}\right)}^{2}\sin\theta_{t,1}-36\pi \tau_{s}{}^{4}R_{T}{}^{2}B_{p}{}^{2}}{6\pi \tau_{s}{}^{2}E^{*}{}^{2}R_{T}B_{p}\left(2R_{T}-B_{p}\right)},$$

(23) $$\delta_{1}=\frac{F_{N,1}E^{*}{}^{3}{\left(2R_{T}+B_{p}\right)}^{2}\sin\theta_{t,1}-36\pi \tau_{s}{}^{4}R_{T}{}^{2}B_{p}{}^{2}\left(\pi -2\right)}{6\pi \tau_{s}{}^{2}E^{*}{}^{2}R_{T}B_{p}\left(2R_{T}-B_{p}\right)}$$

Similarly, the plastic deformation and total deformation of the extrusion contact between the second guide pad and the hole wall are as follows:

(24) $$\delta_{p,2}=\frac{F_{N,2}E^{*}{}^{3}{\left(2R_{T}-B_{p}\right)}^{2}\sin\theta_{t,2}-36\pi \tau_{s}{}^{4}R_{T}{}^{2}B_{p}{}^{2}}{6\pi \tau_{s}{}^{2}E^{*}{}^{2}R_{T}B_{p}\left(2R_{T}-B_{p}\right)}$$

(25) $$\delta_{2}=\frac{F_{N,2}E^{*}{}^{3}{\left(2R_{T}+B_{p}\right)}^{2}\sin\theta_{t,2}-36\pi \tau_{s}{}^{4}R_{T}{}^{2}B_{p}{}^{2}\left(\pi -2\right)}{6\pi \tau_{s}{}^{2}E^{*}{}^{2}R_{T}B_{p}\left(2R_{T}-B_{p}\right)}$$

Finally, Equations (18) and (19) and Equations (22)–(25) can be substituted into Equation (3), and then the positive force of the guide pad under different drilling conditions can be obtained with the help of the drilling force model established in [28]. Consequently, the hole diameter for BTA deep hole machining can be predicted.

## 3. Simulation

### 3.1. FEM Model

Using ABAQUS software, an FEM model of extrusion contact was established between the guide pads and the hole wall of BTA deep hole drilling. The parameters of the BTA drill are shown in Table 1. The BTA drill was connected with the drilling tube, the drill was in contact with the annular hole wall, and the teeth were applied to the cutting force, such that the drilling tube was elastically deformed, and the guide pad was pressed against the hole wall to produce elastic–plastic extrusion. Then, the extrusion contact between the guide pad and the hole wall for BTA deep hole machining was simulated.

The workpiece was a ring simulating the hole wall from tooth cutting. The inner diameter of the workpiece was the same as the outer diameter of the BTA drill, the wall thickness was 10 mm, and the height was 12 mm, which could not only reduce the calculation, but also completely cover the contact area between the guide pads and the hole wall in the axial direction. The workpiece material was pressure vessel steel SA-5083, with the constitutive relation described by the J–C model [29], and the model parameters were as shown in Table 2.

**Figure 7:**
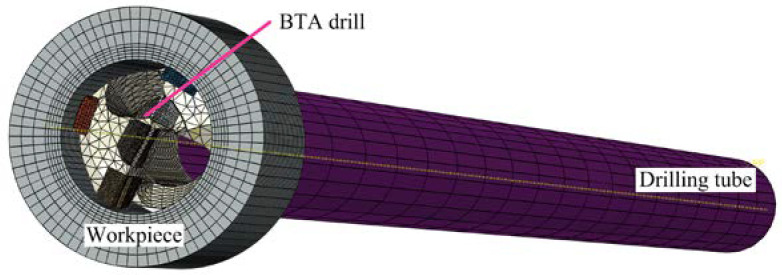
Mesh division of the extrusion model of guide pad and hole wall of BTA deep hole drill.

The drilling tube was set with an elastic body, the BTA drill was set with a rigid body, and the workpiece was set with a plastic body. The key part of the simulation of the contact between the guide pad and the hole wall was the workpiece with a regular shape. Accordingly, the guide pads and drilling tube were meshed with hexahedral and C3D8R first-order reduced integration elements, and the drill body and the teeth with complex structures were meshed with tetrahedral and C3D4 first-order elements. The inner wall area of the ring workpiece and the guide pads were treated with mesh refinement, and the drilling tube was divided using a large mesh. The mesh division of the simulation model is shown in Figure 7.

As a consequence of the BTA drill teeth, guide pads, and body all being welded and connected, and the drill and the drilling tube being connected by threaded connections, each component of the tool system was set to be in binding contact to make it a whole. The workpiece was fixed, and the radial constraint displacement of the end of the drilling tube was zero, which rotated and fed around the *Z*-axis.

The cutting force generated by tooth cutting was applied to the cutting edges of the drill. The sliding friction between the guide pads and the hole wall was added to the guide pads; the circumferential friction coefficient was 0.398, and the axial friction coefficient was 0.0964 [28].

### 3.2. Contact Inclination Angle

**Figure 8:**
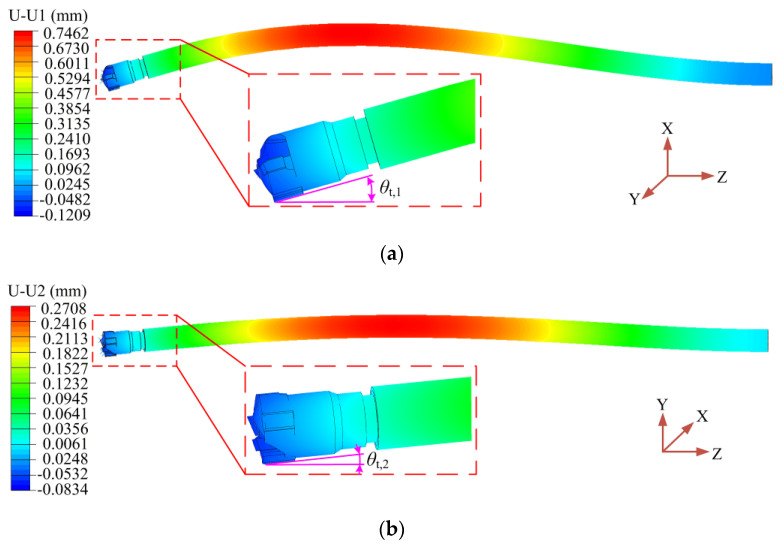
The displacement nephogram of the drilling tube deformation during BTA deep hole machining: (**a**) along the first guide pad; (**b**) along the second guide pad.

The FEM model was established to simulate the bending elastic deformation of the drilling tube after the BTA drill was loaded, and the contact inclination angle between the two guide pads and the hole wall was obtained. By loading the cutting force of the teeth with the drilling speed *n* = 1200 r/min and the drilling feed *f* = 0.08 mm/r, when the overhang length of the drilling tube was *L*_b_ = 500 mm, the deformation displacement nephogram of the drilling tube along the contact axis of the two guide pads and the hole wall was obtained, as shown in Figure 8. The deformation displacement nephogram was the result of enlarging the simulation deformation by 40 times. The maximum displacement of the drilling tube bending deformation along the first guide pad was 2.76 times that along the second guide pad.

The drilling tube length in the FEM model was the tube overhang *L*_b_, and the process parameter used in deep hole machining was the drilling depth *L*_d_. The closest constraint point of the drilling tube to the drilling area was the sealed end of the cutting fluid supply apparatus. Therefore, the drilling depth was equal to the overhang length of the drilling tube minus the length of 300 mm between the support point of the cutting fluid supply apparatus and the contact point of the workpiece.

**Figure 9:**
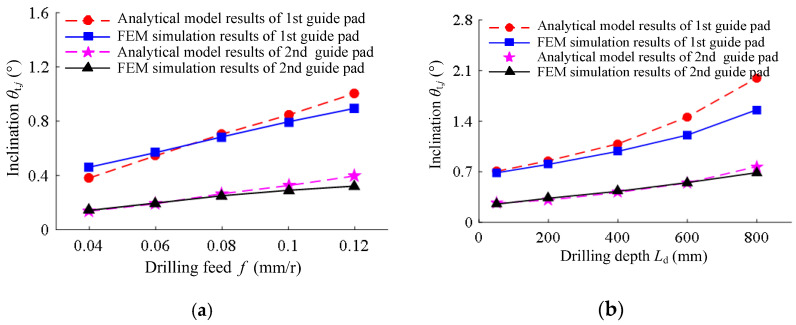
The contact inclination angle from the analytical model results and FEM simulation results: (**a**) under different drilling feeds of drilling speed *n* = 1200 r/min and drilling depth *L*_d_ = 50 mm; (**b**) under different drilling depths of drilling speed *n* = 1200 r/min and feed rate *f* = 0.08 mm/r.

The analytical model results and FEM simulation results of the inclination angle of the extrusion contact between the two guide pads and the hole wall are shown in Figure 9. The inclination angle of the extrusion contact between the two guide pads increased with the increase in drilling feed. With the increase in drilling depth, the contact inclination angle increased, and the growth rate also increased. Under different drilling conditions, the analytical model results of the guide pad inclination angle were basically consistent with the FEM simulation results, while the analytical value and the simulation value had a large error when the drilling depth was *L*_d_ = 800 mm. The main reason for the error is that there was no radial constraint on the connecting end of the drill in the analytical model, whereas in the FEM simulation, when the guide pads extruded the hole wall, the BTA drill was constrained by the radial direction of the workpiece, and the bending deformation and displacement of the drilling tube were reduced. Therefore, the analytical model results of the inclination angle of the guide pads were slightly larger than the FEM simulation results.

### 3.3. Contact Stress

The contact stress nephograms visually display the contact area and contact stress between the guide pads and the hole wall during the BTA deep hole drilling process. The contact area reflects the wear area of the guide pad, while the contact stress reflects the degree of the wear. Under different drilling conditions, the stress nephograms of the extrusion contact between the two guide pads and the hole wall were as shown in Table 3.

The contact areas of the first guide pad were mainly located at the top and upper left areas of the guide pad, while the contact areas of the second guide pad were mainly located at the top, upper right, and left areas of the guide pad. The maximum stress of the contact area between the guide pads and the hole wall increased with the increase in drilling feed and increase in drilling depth. The area of the stress concentration region increased with the increase in drilling feed, but decreased with the increase in drilling depth.

### 3.4. Contact Deformation

**Figure 10:**
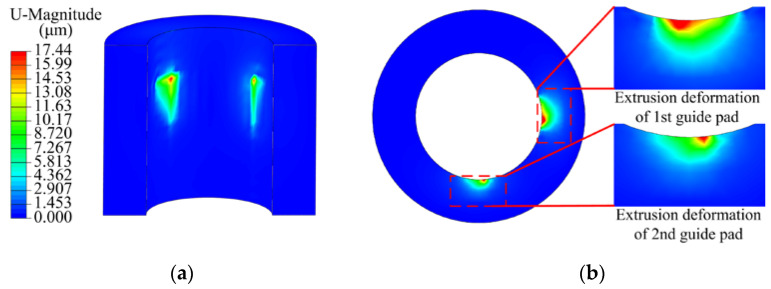
Extrusion deformation nephogram between the guide pads and hole wall: (**a**) cutting plane along workpiece axis; (**b**) cutting plane perpendicular to workpiece axis.

The extrusion of the guide pad to the hole wall changed from contact to elastic deformation, and then from the elastic limit to plastic deformation. When the drill was unloaded, the deformation of the workpiece hole wall was plastic deformation. When the teeth and guide pads were loaded with the tooth cutting force and the guide pad friction force, with drilling speed *n* = 1200 r/min and feed rate *f* = 0.08 mm/r, and then unloaded after a loading time of 0.5 s, with drilling depth *L*_d_ = 50 mm, the deformation nephogram of the workpiece hole wall was as shown in Figure 10. The maximum extrusion deformation of the two guide pads on the hole wall was located in the contact stress concentration area, and the extrusion plastic deformation of the first guide pad was greater than that of the second guide pad. Compared with the second guide pad, the range of the influence area produced by the first guide pad after the hole wall was squeezed, and the deformation was larger.

**Figure 11:**
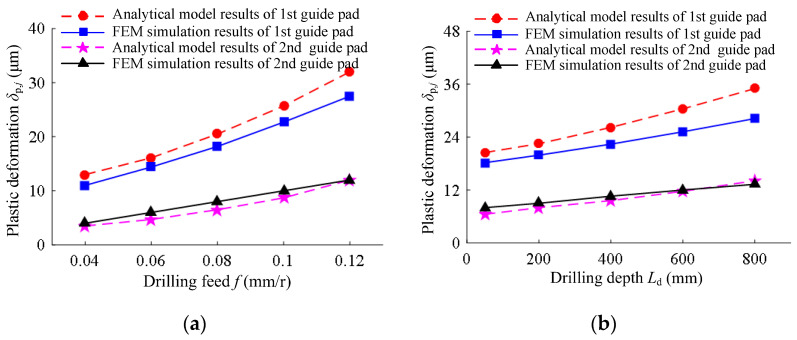
Plastic deformation according to the analytical model results and FEM simulation results: (**a**) under different drilling feeds of drilling speed *n* = 1200 r/min and drilling depth *L*_d_ = 50 mm; (**b**) under different drilling depths of drilling speed *n* = 1200 r/min and feed rate *f* = 0.08 mm/r.

Under different drilling conditions, the analytical model results and finite element simulation results of the plastic deformation caused by the extrusion of the two guide pads to the hole wall were as shown in Figure 11. The variation of the plastic deformation of the two guide pads to the hole wall under different drilling feeds and drilling depths was basically the same. The plastic deformation caused by the extrusion of the first guide pad was about 2.43 times that of the second guide pad. The plastic deformation of the hole wall increased with the increase in drilling feed and drilling depth.

The plastic deformation of the hole wall extrusion increased with the increase in drilling feed and drilling depth. With the increase in drilling feed, the positive pressure on the guide pad increased, and the total extrusion deformation of the guide pad to the hole wall increased, while the elastic deformation of the hole wall springback was constant; thus, the plastic deformation increased. When the drilling depth increased, the suspended length of the drilling tube increased, which led to an increase in the contact angle between the guide pad and the hole wall, a reduction in the extrusion contact area, an increase in the total extrusion deformation, and an increase in the plastic deformation of the hole wall.

## 4. Verification and Discussion

### 4.1. Experimental Equipment

**Figure 12:**
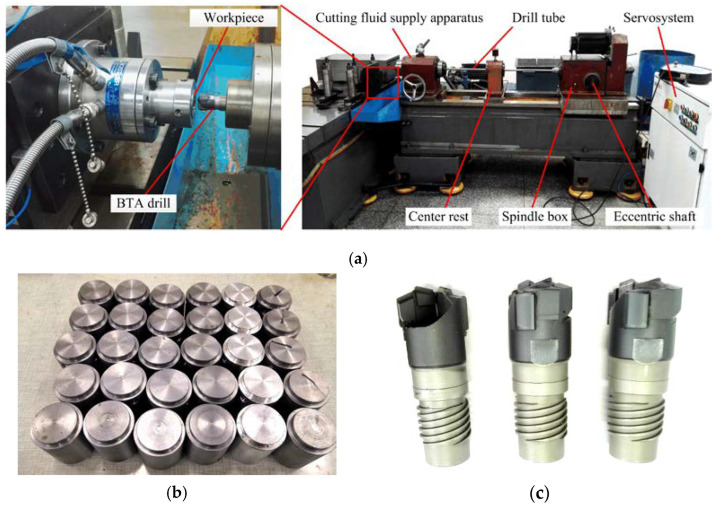
Equipment for deep hole machining tests: (**a**) deep hole machining machine; (**b**) workpiece; (**c**) ∅17.75 BTA drill.

The drilling test used an internal chip removal BTA deep hole drilling machine; the experimental equipment is shown in Figure 12. The length of the drilling tube was 1500 mm, the maximum drilling depth was 1000 mm, and the position accuracy was 0.001 mm. The dynamic viscosity of the cutting fluid was 1.33 × 10^−2^ Pa·s, the maximum supply pressure was 6 MPa, and the flow rate was 90 L/min. The workpiece was a third-generation nuclear power steam generator tube sheet, the material was pressure vessel steel SA-5083, the size was ∅32 × 50 mm, and the drilling depth was 30 mm. The tool was a ∅17.75 mm BTA drill with welded carbide teeth.

### 4.2. Model Validation

During the deep hole machining test of the BTA drill, the wear morphology of the two guide pads under different drilling conditions was as shown in Table 4. The actual wear areas of the two guide pads were very small and concentrated on the top of the guide pads. The first guide pad was more seriously worn than the second guide pad, which is in good agreement with the simulation analysis results of the stress nephograms in Table 3. The extrusion stress concentration area between the guide pad and the hole wall was consistent with the actual wear area of the drill, under different drilling conditions, and the maximum extrusion stress between the guide pad and the hole wall was greater for the first guide strip than the second guide strip, indicating serious wear. Furthermore, the two guide pads were subject to the positive pressure of the hole wall and the friction force in the opposite direction of the relative movement of BTA deep hole drilling; therefore, the actual wear area of the second guide pad also existed along the left edge.

**Figure 13:**
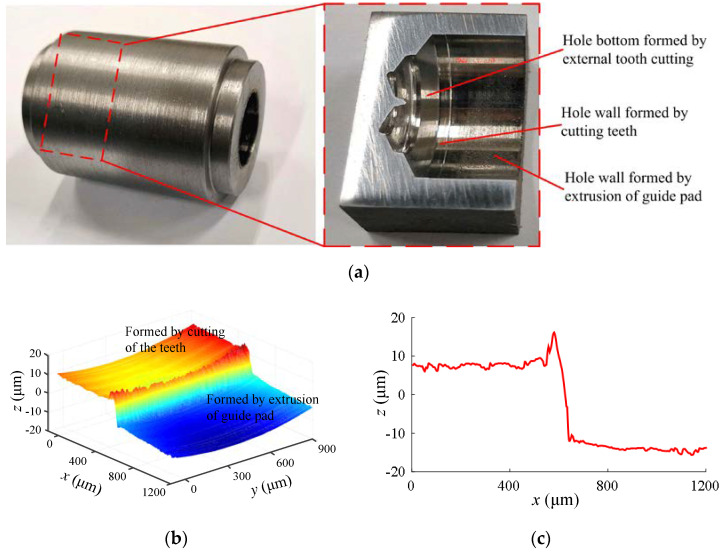
Measurement of total plastic deformation of guide pads and hole wall: (**a**) section piece of deep hole drilling specimen; (**b**) 3D morphology of hole wall deformation zone; (**c**) extraction of total extrusion deformation.

Since the two guide pads extruded the hole wall in sequence at almost the same height along the axis of the drill body, the plastic deformation caused by the extrusion of the first and second guide pads to the hole wall could not be directly measured. The deep hole machined specimens were cut by wire cutting, and the tomographic scanning and reconstruction of the tooth cutting area, the guide pad burnishing area, and the transition area of the hole wall were performed using a Leica DCM 3D laser confocal microscope, thus extracting the three-dimensional shape of the area. The total plastic deformation of the hole wall was obtained using the height difference between the tooth cutting area and the guide pad burnishing area, where *δ*_pc_ = *δ*_p,1_ + *δ*_p,2_. The measurement method of the total plastic deformation of the hole wall is shown in Figure 13.

In addition, the roughness Ra along the axial direction of the hole wall was calculated using the extracted three-dimensional shape data. The results show that the roughness of the hole wall after the extrusion of the guide pads was about 0.2 to 0.5 times that of the hole wall formed by tooth cutting. The extrusion contact between the guide pads and the hole wall not only expanded the hole diameter, but also reduced the roughness of the hole wall and improved the surface quality of the hole wall, which is consistent with the qualitative analysis results in [5,16].

**Figure 14:**
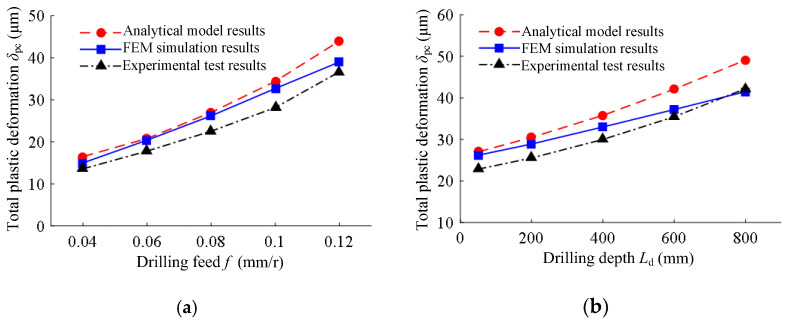
The total plastic deformation of the hole wall under different drilling conditions: (**a**) under different drilling feeds of drilling speed *n* = 1200 r/min and drilling depth *L*_d_ = 50 mm; (**b**) under different drilling depths of drilling speed *n* = 1200 r/min and feed rate *f* = 0.08 mm/r.

The analytical model results, FEM simulation results, and experimental test results of the total plastic deformation of the hole wall under different drilling conditions are shown in Figure 14. The analytical model results, FEM simulation results, and experimental test results of the total plastic deformation of the guide pad extruding the hole wall were consistent. With the increase in drilling feed, the total plastic deformation increased, and, as the drilling depth increased, the total plastic deformation increased. The maximum error between the FEM simulation results and the test results was 18.1%, and the maximum error between the analytical model results and the test results was 23.6%, indicating that the established FEM simulation model of the extrusion contact between the guide pad and the hole wall was correct, and the established analytical model was reliable, thereby accurately predicting the extrusion deformation of the guide pad to the hole wall during BTA deep hole drilling.

**Figure 15:**
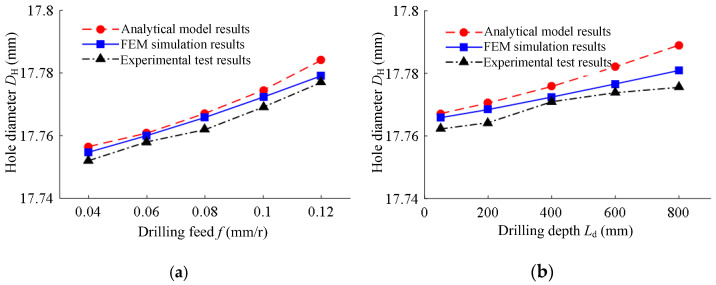
The hole diameters under different drilling conditions: (**a**) under different drilling feeds of drilling speed *n* = 1200 r/min and drilling depth *L*_d_ = 50 mm; (**b**) under different drilling depths of drilling speed *n* = 1200 r/min and feed rate *f* = 0.08 mm/r.

The hole diameters of BTA self-guiding deep hole machining from the analytical model, FEM simulation, and experimental test under different drilling feeds and drilling depths are shown in Figure 15. The total extrusion deformation between the guide pad and the hole wall was between 10 and 50 μm. With the increase in drilling feed, the machining hole diameter increased; with the increase in drilling depth, the machining hole diameter increased. The analytical model results, FEM simulation results, and experimental test results of the hole diameter under different drilling feeds and drilling depths were basically consistent. This shows that the established prediction model of the hole diameter of BTA deep hole drilling was correct and could accurately predict the diameter of deep hole machining.

## 5. Conclusions

In this paper, the self-guiding hole forming mechanism of BTA deep hole machining was revealed, and a novel extrusion contact model between the guide pad and the hole wall and a new hole diameter prediction model were established, while the variation laws of the contact inclination angle, contact stress, extrusion deformation, and machining hole diameter with drilling parameters were obtained. The conclusions below were drawn.

The guide pads were in extrusion contact with the hole wall in a small area, and there was a serious concentration of extrusion stress. The areas where the extrusion stress was concentrated were located at the top of the guide pad near the teeth. The extrusion stress concentration area of the guide pad was consistent with the actual wear area of the BTA drill.

The variation of the plastic deformation of the two guide pads to the hole wall was basically the same under different drilling feeds and drilling depths. The plastic deformation produced by the extrusion of the first guide pad was about 2.43 times that of the second guide pad. The extrusion plastic deformation of the guide pad to the hole wall increased with the increase in drilling feed and drilling depth.

The total extrusion deformation between the guide pad and the hole wall was between 10 and 50 μm. The maximum error between the FEM simulation results and the test results was 18.1%, and the maximum error between the analytical model results and the test results was 23.6%. The analytical model results, FEM simulation results, and experimental test results of the total extrusion plastic deformation of the guide pad to the hole wall and the machining hole diameter were consistent, and the established analytical and simulation models of the extrusion contact of the guides pad and the hole wall were correct. Thus, the accurate prediction of the extrusion deformation of the guide pads and the hole wall, as well as the machining hole diameter, during the BTA deep hole drilling could be realized.

As deep hole machining is affected by the poor rigidity of the machining system, the drilling process is affected by the vibration of the tool system, and the tool system is always in a high-pressure cutting fluid. If the dynamic characteristics of the tool system and the influence of cutting fluid can be considered in the future, the prediction accuracy of the model can be further improved.

## Figures and Tables

**Table 1:** Geometric parameters of ∅17.75 mm BTA drill.

Teeth	Rake Angle	Approach Angle	Edge Angle	Tooth Width	Guide Pads	Position Angle	Length	Width	Material
Central tooth	−5°	15°	5°	5 mm	1st guide pad	87°	9 mm	5 mm	M20
Intermediate tooth	0°	18°	0°	3.5 mm	2nd guide pad	183°	9 mm	5 mm	M20
External tooth	0°	18°	0°	4 mm					

**Table 2:** J–C constitutive model parameters of workpiece SA-5083.

Material	Density *ρ* (kg/m^3^)	Elastic Modulus E (GPa)	Poisson’s Ratio *ε*	Yield Strength *A* (MPa)	Work Hardening *B* (MPa)	Hardening Coefficient *n*	Strain Rate Constant *C*	Thermal Softening *m*	Melting Temperature *T_m_* (°C)
SA-5083	7920	210	0.269	885	450	0.45	0.1	1.02	1985

**Table 3:** Extrusion stress nephograms between the guide pads and hole wall under different drilling parameters.

Figure 1	1st Guide Pad	2nd Guide Pad	Depth *L*_d_ (mm)	1st Guide Pad	2nd Guide Pad
0.04	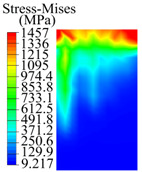	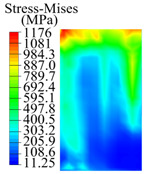	50	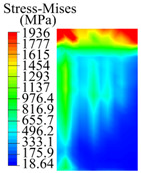	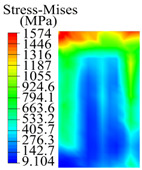
0.06	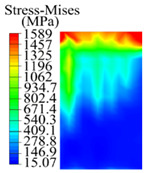	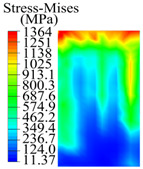	200	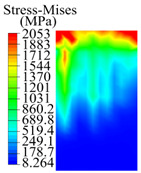	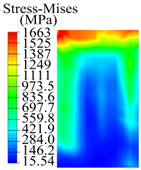
0.08	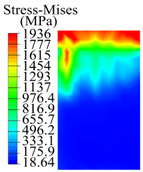	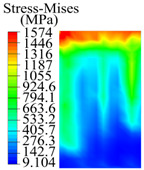	400	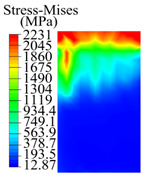	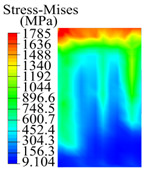
0.1	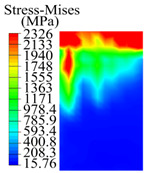	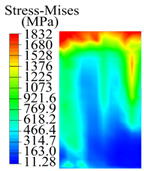	600	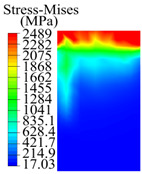	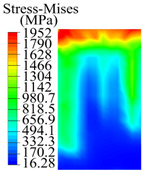
0.12	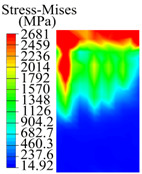	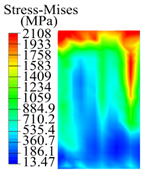	800	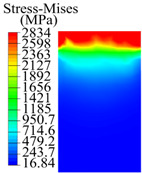	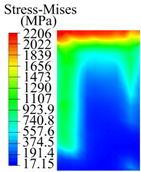

**Table 4:** Wear morphology of the guide pads of the BTA drill under different drilling conditions.

BTA Drill	No. 1 Tool	No. 2 Tool	No. 3 Tool	No. 4 Tool
1st guide pad	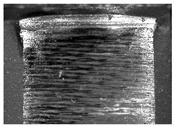	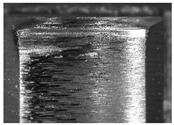	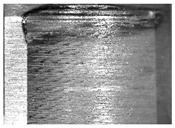	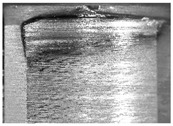
2nd guide pad	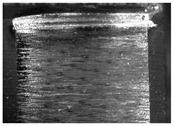	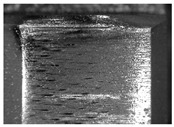	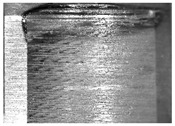	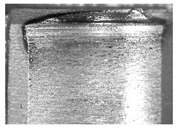

## Data Availability

Not applicable.

## References

[1] Si Y., Kong L., Chin J.-H., Guo W., Wang Q. (2021). Whirling detection in deep hole drilling process based on multivariate synchrosqueezing transform of orthogonal dual-channel vibration signals. Mech. Syst. Signal Process..

[2] Feng Y., Zheng H., Han X., Liu Z. (2022). Multiobjective Optimization of Cutting Parameters for TA10 Alloy Deep-Hole Drilling. Materials.

[3] Lew M., Chaudhari A., Neo W.K., Kumar S., Amrun M., Rashid M.A., Rahman M. (2020). Modeling of dynamic behavior of multispan gundrilling shaft with coolant and its effect on straightness deviation. CIRP J. Manuf. Sci. Technol..

[4] Li X.-B., Zheng J.-M., Li Y., Kong L.-F., Shi W.-C., Guo B. (2019). Investigation of Chip Deformation and Breaking with a Staggered Teeth BTA Tool in Deep Hole Drilling. Metals.

[5] Zhang H., Shen X., Bo A., Li Y., Zhan H., Gu Y. (2017). A multiscale evaluation of the surface integrity in boring trepanning association deep hole drilling. Int. J. Mach. Tools Manuf..

[6] Haddag B., Nouari M., Moufki A. (2019). Experimental analysis of the BTA deep drilling and a new analytical thermomechanical model for assessment of cutting forces and BTA drill design. Int. J. Adv. Manuf. Technol..

[7] Malarvizhi S., Chaudhari A., Woon K.S., Kumar A.S., Rahman M. (2016). Influence of Burnishing Axial Interference on Hole Surface Quality in Deep Hole Drilling of Inconel 718. Procedia Manuf..

[8] Bleicher F., Reiter M., Brier J. (2019). Increase of chip removal rate in single-lip deep hole drilling at small diameters by low-frequency vibration support. CIRP Ann..

[9] Ahmed A., Lew M., Diwakar P., Kumar A.S., Rahman M. (2019). A novel approach in high performance deep hole drilling of Inconel 718. Precis. Eng..

[10] Yu D. (2017). Self-centering positioner and principle for locating and guiding deep-hole drills using oil films. Int. J. Adv. Manuf. Technol..

[11] Si Y., Zhang Z., Kong L., Zheng J. (2021). Condition monitoring of deep-hole drilling process based on improved empirical wavelet de-noising and high multiple frequency components of rotation frequency. Int. J. Adv. Manuf. Technol..

[12] Kong L., Cao S., Chin J.-H., Si Y., Miao F., Li Y. (2020). Vibration suppression of drilling tool system during deep-hole drilling process using independence mode space control. Int. J. Mach. Tools Manuf..

[13] Tnay G., Wan S., Woon K., Yeo S. (2016). The effects of dub-off angle on chip evacuation in single-lip deep hole gun drilling. Int. J. Mach. Tools Manuf..

[14] Zhang X., Tnay G.L., Liu K., Kumar A.S. (2018). Effect of apex offset inconsistency on hole straightness deviation in deep hole gun drilling of Inconel 718. Int. J. Mach. Tools Manuf..

[15] Richardson R., Bhatti R. (2001). A review of research into the role of guide pads in BTA deep-hole machining. J. Mater. Process. Technol..

[16] Biermann D., Bleicher F., Heisel U., Klocke F., Möhring H.-C., Shih A. (2018). Deep hole drilling. CIRP Ann..

[17] Woon K., Chaudhari A., Rahman M., Wan S., Kumar A.S. (2014). The effects of tool edge radius on drill deflection and hole misalignment in deep hole gundrilling of Inconel-718. CIRP Ann..

[18] Zhang H., Shen X., Li Y., Yang F., Kwon P., Zhang D., Zi B., Cui G., Ding H. (2016). The Effects of Guide Pads on Bore Diameter Enlargement Magnitude in Deep Hole Drilling. MATEC Web Conf..

[19] Felinks N., Rinschede T., Biermann D., Stangier D., Tillmann W., Fuß M., Abrahams H. (2021). Investigation into deep hole drilling of austenitic steel with advanced tool solutions. Int. J. Adv. Manuf. Technol..

[20] Neo D.W.K., Liu K., Kumar A.S. (2020). High throughput deep-hole drilling of Inconel 718 using PCBN gun drill. J. Manuf. Process..

[21] Yang S., Tong X., Ma X., Ji W., Liu X., Zhang Y. (2018). The guide block structure design of boring and trepanning association (BTA) deep hole drilling. Int. J. Adv. Manuf. Technol..

[22] Astakhov V., Osman M. (1996). An analytical evaluation of the cutting forces in self-piloting drilling using the model of shear zone with parallel boundaries. Part 1: Theory. Int. J. Mach. Tools Manuf..

[23] Astakhov V., Osman M. (1996). An analytical evaluation of the cutting forces in self-piloting drilling using the model of shear zone with parallel boundaries. Part 2: Application. Int. J. Mach. Tools Manuf..

[24] Matsuzaki K., Ryu T., Sueoka A., Tsukamoto K. (2015). Theoretical and experimental study on rifling mark generating phenomena in BTA deep hole drilling process (generating mechanism and countermeasure). Int. J. Mach. Tools Manuf..

[25] Sakuma K., Taguchi K., Katsuki A. (1980). Study on Deep-hole-drilling with Solid-boring Tool: The Burnishing Action of Guide Pads and Their Influence on Hole Accuracies. Bull. JSME.

[26] Wang L., Sun X., Huang Y. (2007). Friction analysis of microcosmic elastic-plastic contact for extrusion forming. J. Mater. Process. Technol..

[27] Song Z., Komvopoulos K. (2013). Elastic–plastic spherical indentation: Deformation regimes, evolution of plasticity, and hardening effect. Mech. Mater..

[28] Li X., Zheng J., Li Y., Kong L., Shi W., Guo B. (2018). Modeling and Distribution Laws of Drilling Force for Staggered Teeth BTA Deep Hole Drill. Math. Probl. Eng..

[29] Li L., He N., Hao X., Yang Y. (2019). Deep-hole gun drilling mechanics model of Ti6Al4V alloy based on Johnson and Cook flow stress model. Int. J. Adv. Manuf. Technol..

